# Hypomagnesaemia and its determinants in a contemporary primary care cohort of persons with type 2 diabetes

**DOI:** 10.1007/s12020-019-02116-3

**Published:** 2019-10-24

**Authors:** Femke Waanders, Robin P. F. Dullaart, Michel J. Vos, Steven H. Hendriks, Harry van Goor, Henk J. G. Bilo, Peter R. van Dijk

**Affiliations:** 1 Department of Nephrology, Isala, Zwolle, The Netherlands; 2 Department of Endocrinology, University Medical Center, University of Groningen, Groningen, The Netherlands; 3 Department of Clinical Chemistry, Isala, Zwolle, The Netherlands; 4 Department of General practice, University of Leiden, Leiden, The Netherlands; 5 Department of Pathology and Medical Biology, University Medical Center, University of Groningen, Groningen, The Netherlands; 6 Department of Internal Medicine, University Medical Center, University of Groningen, Groningen, The Netherlands; 7 Diabetes Centre, Isala, Zwolle, The Netherlands

**Keywords:** Type 2 diabetes, Magnesium, Hypomagnesaemia, Prevalence

## Abstract

**Aims:**

Among persons with type 2 diabetes mellitus (T2DM) hypomagnesaemia has been reported in 14–48% of patients. This may be of significance given the emerging associations of hypomagnesaemia with glucometabolic disturbances and possibly even complications. We assessed the prevalence of hypomagnesaemia and its determinants, in a well-defined cohort of persons with T2DM treated in primary care.

**Methods:**

Observational cohort study among persons with T2DM treated in primary care in the Northeast of the Netherlands. Magnesium was measured using a colorimetric endpoint assay (Roche). Hypomagnesaemia was defined as a serum magnesium level <0.70 mmol/L. Pearson correlations were performed to correlate variables with serum magnesium. Next, a stepwise backward regression model was made.

**Results:**

Data of 929 persons (55% male) with a mean age of 65 (± 10) years, diabetes duration 6.5 [3.0–10.1] years, and HbA1c concentration 6.7 (± 0.7)% (50 (± 9) mmol/mol) were analysed. Serum magnesium was 0.79 (± 0.08) mmol/L. The percentage of persons with magnesium deficiency was 9.6%. Age, diabetes duration, BMI, HbA1c, use of metformin, sulfonylurea derivatives, and DPP4 inhibitors were negatively associated with magnesium concentrations. In contrast, LDL cholesterol and serum creatinine were positively associated serum magnesium.

**Conclusions:**

Hypomagnesaemia was present in 9.6% of T2DM patients treated in primary care. This percentage is remarkably lower than reported previously, possibly due to the unselected nature of our population. Concerning T2DM-related factors, only BMI, HbA1c and the use of metformin, sulfonylurea derivatives and DPP4 inhibitors correlated negatively with magnesium concentrations.

## Introduction

Over the past decades, the relevance of abnormalities in magnesium metabolism among persons with type 2 diabetes mellitus (T2DM) is increasingly appreciated [1]. Low concentrations of magnesium in persons with T2DM have been attributed to (poor) dietary intake, autonomic dysfunction, altered insulin metabolism, glomerular hyperfiltration, osmotic diuresis, metabolic acidosis, hypophosphataemia, and hypokalaemia [2]. Low magnesium concentrations are also linked to poor glycaemic control and the presence of micro- and macrovascular complications. Moreover, plasma magnesium was found to predict the risk of incident T2DM in a large cohort from the north of the Netherlands [3]. Of note, supplementation of magnesium is easy, low-cost and was shown to improve insulin sensitivity [4], glucose metabolism [5, 6] and to retard progression from pre-diabetes to overt diabetes [7].

Hypomagnesaemia in persons with T2DM has been reported to vary between 14 and 48% [2]. However, most of these studies were performed within selected populations [8], under clinical circumstances [9, 10], included both adults and children [10], included a relatively low number of participants [9, 11, 12] or included participants treated in a secondary or tertiary care setting [13]. In addition, some of these studies were performed over 20 years ago and did not take the influence of novel treatment options for DM and associated conditions (i.e. DPP4 inhibitors or statins) into account.

Given the increasing incidence of DM, the potential importance of disturbances in magnesium metabolism and potential for supplementation we aimed to investigate the prevalence of hypomagnesaemia in a well-characterised, but unselected cohort of outpatients with T2DM treated in primary care. In addition, determinants associated with serum magnesium concentrations were identified.

## Patients and methods

Clinical data and blood samples were obtained from the prospective, observational cohort e-VitaDM study, which was designed to assess the feasibility of using an online platform in routine primary healthcare for subjects with T2DM. As a pre-specified part of the e-VitaDM study, participants were assessed in a long-term follow-up protocol. This prospective arm was nested within the Zwolle Outpatient Diabetes project Integrating Available Care (ZODIAC) study. Both the e-VitaDM and the ZODIAC study are described in detail elsewhere [14]. Primary aim of the present study was to investigate the prevalence of hypomagnesaemia in T2DM outpatients. As secondary outcomes the cross-sectional associations between baseline concentrations of magnesium with indices of T2DM management, including HbA1c and the presence of complications, were assessed.

This study was conducted in general practices that are connected to the Care Group Drenthe in the Drenthe-region (located in the North-East) of the Netherlands: 42 out of the 110 general practices in this care group agreed to participate. In these practices, ~8300 persons were treated for their T2DM between 2012 and 2014. Participants were recruited during a regular check-up by their practice nurse and included from May 2012 until September 2014 [14, 15]. Persons with T2DM, aged ≥18 years and the general practitioner as main care provider for T2DM were eligible for participation [14]. Only patients who were co-treated in secondary care (for their DM), who were terminally ill or had dementia were excluded. A total of 1710 out of 3988 participants, who were asked to participate in the eVita-DM study, gave written informed consent. Of these persons, 764 were not included in the present study due to the inability to match blood samples taken in the e-VitaDM with the clinical data from the ZODIAC study. A further 17 individuals were excluded from the final analysis because of insufficient blood material to perform the measurements. Consequently, the final study cohort consisted of 929 participants.

Baseline demographic data included age, gender, diabetes duration, body mass index (BMI), alcohol intake and smoking habits. Information concerning alcohol intake and smoking habits was derived from questionnaires at baseline. Medical data were extracted from the diabetes-specific database at the Diabetes Centre of the Isala hospital (Zwolle, The Netherlands). This centre gathers data of primary care-treated persons with T2DM in a large part of the Netherlands on an annual basis, to provide benchmark information to general practitioners. This database includes information on physical examination, use of medication, and laboratory blood and urine tests. The following data were extracted: date of diabetes diagnosis, height, weight, diastolic and systolic blood pressure, cholesterol, HbA1c, estimated glomerular filtration rate based on the modification of diet in renal disease (MDRD) equation, urine creatinine, urine albumin, urine albumin:creatinine ratio, and the presence of macrovascular- and microvascular complications.

Macrovascular complications were defined as (a history of) angina pectoris, myocardial infarction, percutaneous transluminal coronary angioplasty, coronary artery bypass grafting, cerebrovascular accident or transient ischaemic attack. Microvascular complications were defined as diabetic retinopathy, albuminuria (both micro- and macroalbuminuria) and diabetic peripheral neuropathy. Microalbuminuria was defined as 20–200 mg/L albumin or an albumin:creatinine ratio between 2.5–25 mg/mmol in men and 3.5–35 mg/mmol in women. Macro-albuminuria was defined as >200 mg/L albumin or a albumin:creatinine ratio >25 mg/mmol and 35 mg/mmol for men and women, respectively [16]. An ophthalmologist determined the presence of diabetic retinopathy biannually. Foot sensibility was tested with 5.07 Semmes–Weinstein monofilaments. Neuropathy was defined as two or more errors in a test of three, affecting at least one foot. Blood glucose-lowering therapy was categorised into: dietary measures only, oral blood glucose lowering drugs (OBGLDs) including metformin, sulfonylurea derivatives, thiazolidinediones and DDP4 inhibitors, and insulin therapy. HbA1c is expressed in both IFCC-recommended (as mmol/mol) and DCCT-derived (as %) units.

Participants were instructed to be in a fasting state when the blood samples were collected. Venous blood samples were collected into BD Vacutainer^TM^ serum tubes, centrifuged and the serum was stored directly in aliquots at −80 °C until measurement. Serum magnesium, total cholesterol, HDL cholesterol, triglycerides and creatinine (enzymatic method) were measured on a Modular Analytics P-module (Roche Diagnostics, Mannheim, Germany). LDL cholesterol was quantitated indirectly using the Friedewald formula [17]. A magnesium deficiency was defined as serum concentration <0.7 mmol/L [1, 13]. Tosoh G7 standard mode was used to measure HbA1c.

Normally distributed data are presented as mean ± SD and non-normally distributed data are presented as median [interquartile range]. Normality of variables was assessed using frequency distribution histograms and Q–Q plots. Variables with a non-parametric distribution were log_10_ transformed before analysis. Univariable analysis for correlation, using the Pearson correlation coefficient, was performed to investigate the association between serum magnesium and other variables. Variables with a *p* value <0.1, not corrected for multiple testing, were checked for confounding by performing partial regression analyses. Next, multivariable linear regression analysis was performed to investigate associations between serum magnesium dependent variable and multiple independent covariates. In this stepwise backward regression model, variables with *p* > 0.1 were eliminated. Covariates were used in the multivariable model based on previous literature (diabetes duration, HbA1c, creatinine, use of metformin, insulin, beta-blockers, diuretics [2, 13, 18]) or in case the *p* value was ≤0.1 in the univariable analysis. The model was checked for collinearity. A two-sided *p* value <0.05 was considered statistically significant. All statistical analyses were performed using SPSS version 24 (IBM, USA).

The study protocol was registered prior to the start of the study (study ID METC 11.10117) and approved by the Medical Ethical Committee of Isala (Zwolle, the Netherlands). The protocol was also registered on clinicaltrials.gov (study ID NCT01570140). All participants gave written informed consent.

## Results

Baseline characteristics of the 929 persons with T2DM are presented in Table 1. The cohort had a mean age of 65 (±10) years, 55% were male, diabetes duration was 6.5 [3.0–10.1] years, and HbA1c concentration was 6.7 ± 0.7 % (50 ± 9 mmol/mol). At baseline, 31% of the participants had a microvascular complication and 25% had a macrovascular complication. Concerning treatment, 20% of the participants were treated with diet alone, while 78% and 13% of the participants were treated with OBGLD and/or insulin, respectively.

**Table 1:** Baseline characteristics and univariable analysis of correlation with magnesium

	Overall *N* = 931	Univariable Pearson correlation coefficient	*P*-value
**Demographics**			
Age, years	65.1 ± 10.1	−0.092	**0.005**
Diabetes duration, years	6.5 [3.0–10.1]	−0.207	**<0.001**
Male gender, *n* (%)	508 (54.7)	0.045	0.169
Current smoker, *n* (%)	154 (16.4)	0.035	0.289
BMI, kg/m^2^	29.3 [26.8–33.0]	−0.140	**<0.001**
Systolic blood pressure, mmHg	135.6 [125.0–144.0]	−0.064	0.054
Diastolic blood pressure, mmHg	80.0 [71.0–83.0]	0.009	0.777
**Complications**			
Microvascular complications, *n* (%)			
Retinopathy, *n* (%)	38 (4.1)	−0.008	0.801
Neuropathy, *n* (%)	171 (18.4)	−0.028	0.401
Microalbuminuria, *n* (%)	116 (12.5)	−0.049	0.133
Macroalbuminuria, *n* (%)	11 (1.2)	−0.012	0.712
Macrovascular events, *n* (%)			
Angina pectoris, *n* (%)	71 (7.8)	−0.036	0.272
Myocardial infarction, *n* (%)	79 (8.5)	−0.043	0.190
PTCA, *n* (%)	25 (2.7)	−0.021	0.525
CABG, *n* (%)	45 (4.8)	−0.033	0.315
TIA, *n* (%)	31 (3.3)	−0.016	0.621
CVA, *n* (%)	55 (5.9)	0.002	0.941
Heart failure, *n* (%)	24 (2.6)	−0.023	0.485
**Laboratory measures**			
HbA1c, mmol/mol (%)	6.7 ± 0.7 (50 ± 9)	−0.181	**<0.001**
eGFR (MDRD), mL/min/1.73 m^2^	73.0 [61.0–86.0]	−0.055	0.094
Total cholesterol, mmol/L	4.4 [3.7–5.0]	0.142	**<0.001**
HDL cholesterol, mmol/L	1.3 [1.0–1.5]	−0.003	0.923
Total cholesterol:HDL ratio	3.4 [2.8–4.2]	0.114	**<0.001**
LDL cholesterol, mmol/L	2.3 [1.8–2.8]	0.166	**<0.001**
Triglycerides, mmol/L	1.5 [1.1–2.1]	0.002	0.941
Creatinine, µmol/L	78.0 [67.5–90.0]	0.104	**0.002**
Urine albumine:creatinine ratio, mg/mmol	0.8 [0.4–1.5]	−0.076	**0.027**
**Treatment**			
Dietary measures only, *n* (%)	181 (19.5)	0.132	**<0.001**
Oral blood glucose lowering drugs, *n* (%)	725 (78.0)		
Metformin, *n* (%)	691 (74.4)	−0.110	**0.001**
Sulfonylurea derivatives, *n* (%)	260 (28.0)	−0.132	**<0.001**
Thiazolinediones, *n* (%)	10 (1.1)	0.021	0.515
DPP4 inhibitors, *n* (%)	35 (3.8)	−0.069	**0.035**
Insulin, *n* (%)	120 (12.9)	−0.100	**0.002**
Antihypertensive drugs, *n* (%)	784 (84.4)		
Diuretics, *n* (%)	348 (37.5)	−0.029	0.389
Beta-blockers, *n* (%)	350 (37.5)	−0.041	0.226
Calcium antagonists, *n* (%)	169 (18.2)	−0.014	0.676
RAAS blockers, n (%)	507 (54.6)	−0.091	**0.007**
Cholesterol lowering drugs, *n* (%)	738 (79.4)		
Statins, *n* (%)	679 (73.1)	−0.061	0.076
Fibrates, *n* (%)	6 (0.6)	0.043	0.213
Thrombocyte aggregation inhibitors, *n* (%)	166 (17.9)	−0.064	0.052

Data are presented as number (%), mean (SD) or median [IQR]. Variables with a non-parametric distribution were log_10_ transformed before calculation of the Pearson correlation coefficient. Significant (*p* < 0.05) values are highlighted in bold. HbA1c is expressed in both IFCC derived mmol/mol as DCCT derived %

*BMI* body mass index, *MI* myocardial infarction, *PTCA* percutaneous transluminal coronary angioplasty, *CABG* coronary artery bypass grafting, *TIA* transient ischaemic attack, *CVA* cerebral vascular event, *HbA1c* glycated haemoglobin, *eGFR* estimated glomerular filtration rate, *HDL* high-density lipoprotein, *LDL* high-density lipoprotein, *DDP4* dipeptidyl peptidase 4, *RAAS* renin-angiotensin-aldosterone system

The mean serum magnesium concentration in the cohort was 0.79 ± 0.08 mmol/L. There were no significant differences in magnesium concentrations between men and women (men: 0.79 ± 0.07 mmol/L: women: 0.79 ± 0.08 mmol/L, *p* = 0.17). Persons with HbA1c concentrations <7.0% (<53 mmol/mol) (*n* = 629) had lower magnesium concentrations as compared to persons with higher HbA1c levels (0.80 ± 0.08 mmol/L vs. 0.77 ± 0.07 mmol/L, *p* < 0.05). Hypomagnesaemia was present in 89 (9.6%) persons. In univariable regression analysis, a negative correlation between serum total magnesium concentrations and age, diabetes duration, BMI, HbA1c, use of metformin, sulfonylurea derivatives, DPP4 inhibitors, insulin and RAAS blocking medication was demonstrated (see Table 1). In contrast there was a positive correlation between magnesium and total cholesterol, total cholesterol:HDL ratio, LDL cholesterol, serum creatinine. There was no correlation of serum magnesium with triglycerides or the presence or absence of complications.

In multivariable regression analyses, age, diabetes duration, BMI, HbA1c, the use of metformin, sulfonylurea derivatives and DPP4 inhibitors were negatively associated with serum magnesium concentrations (see Table 2). On the other hand, LDL cholesterol and creatinine were positively associated with magnesium concentrations.

**Table 2:** Stepwise multivariable regression analysis with baseline total magnesium concentrations as dependent variable

	St. Beta	*P*-value	Partial correlation
Age, years	−0.111	**0.002**	−0.101
Log_10_ Diabetes duration, years	−0.120	**0.001**	−0.110
Log_10_ BMI, kg/m^2^	−0.156	**0.001**	−0.149
HbA1c, %	−0.101	**0.003**	−0.096
Log_10_ LDL cholesterol, mmol/L	0.141	**0.001**	0.138
Log_10_ creatinine	0.109	**0.001**	0.105
Metformin*	−0.077	**0.019**	−0.075
Sulfonylurea derivatives*	−0.085	**0.010**	−0.082
DPP4 inhibitors*	−0.064	**0.047**	−0.063
RAAS blockers*	−0.061	0.057	−0.061
Thrombocyte aggregation inhibitors	−0.062	0.054	−0.061

*No = 0, yes = 1. Significant values are expressed in bold. Adjusted *R*^2^ is 0.128 for the model

## Discussion

In this cohort of 929 persons with T2DM treated in primary care hypomagnesaemia was present in 9.6% of all participants. Increasing age, diabetes duration, BMI, HbA1c and the use of metformin, sulfonylurea derivatives and DPP4 inhibitors were associated with low magnesium concentrations while LDL cholesterol and creatinine were associated with higher concentrations.

Interestingly, the prevalence of hypomagnesaemia found in the present study is considerably lower as compared to previous studies, which reported prevalences varying from 14 to 48% [2]. It is unlikely that the assay system used to determine serum magnesium could account for these differences. In all studies a colorimetric assay was used. Differences in the population studied may account for the differences found. There were barely exclusion criteria for the present study; therefore our results reflect real-life clinical practice. Differences in HbA1c levels could also explain differences, as magnesium is correlated with blood glucose [13, 19, 20]. Unfortunately, not all previous studies reported HbA1c levels. In the study by Kurstjens 30.6% of the patients had a hypomagnesaemia and overall mean HbA1c was 7.9 ± 1.28% (63 ± 14 mmol/L): both are higher as compared with the current study.

Another explanation could be the severity of the T2DM. Most previous studies, that found higher prevalence of hypomagnesaemia as compared with our study, were performed in secondary or even tertiary care. In these studies nearly most patient were poorly regulated, had a mean diabetes duration of >10 years, were treated with blood glucose lowering medication and frequently also used a significant amount of magnesium lowering co-medication. In the current primary care treated population, 19.5% of patients used only dietary measures as T2DM treatment, indicating acceptable glycaemic control, and had a median diabetes duration of 6.5 years. Although our findings may indicate that the magnitude of hypomagnesaemia in T2DM is overestimated by previous clinical studies. Nevertheless, hypomagnesaemia still remain a reason for concern, in particular in the light of data linking low magnesium concentrations to arterial calcification and subsequent cardio-metabolic comorbidity [21, 22]. Remarkably, we did not find an association between serum magnesium concentrations and the presence of macrovascular complications.

In accordance with previous studies we found a negative association between magnesium concentrations and HbA1c. This may be explained by decreased tubular reabsorption caused by hyperglycaemia and/or hyperfiltration [10, 18, 20]. Of further relevance we did not observe a significant association between magnesium and triglycerides. As a post-hoc univariable analysis among participants not using cholesterol-lowering therapy (*n* = 190), we also observed no association between serum magnesium and triglycerides (St. beta 0.033, adjusted *R*^2^ −0.004, *p* = 0.651). This is in contrast to other studies that did find an association among persons with T2DM (20) also independent of waist circumference and BMI [13]. Although the low number of persons may have accounted for the lack of association in the current post-hoc analysis it may also indicate that there is no relevant correlation between magnesium and triglycerides in an unselected Type 2 diabetic population. Likewise, we also found no association of serum magnesium with triglycerides in the PREVEND cohort [18, 23]. The PREVEND cohort consists of persons from the general population living in the Northern part of the Netherlands. Of notice, the prevalence of a hypomagnesaemia (<0.7 mmol/L, measured using Roche Modular colourimetric assay) was 1.3% (72 out of 5568 persons) amongst all persons and 6.6% (18 out of 272 persons) amongst the persons with diabetes (data derived from [3, 18]).

In this cohort the use of DPP4 inhibitors seem to lower magnesium concentrations. To the best of our knowledge, this has not been described before. Based on the working mechanism of DPP4 inhibitors and previous literature, we cannot explain this unexpected finding. On the contrary; it was demonstrated recently that the glucagon-like peptide-1 receptor agonist lixisenatide, which also intervenes on the incretin system, reduces renal magnesium excretion in T2DM subjects. The use of DPP4 inhibitors is a third line treatment option in the Netherlands (after dietary measures, metformin and a sulfonylurea derivative) for patient with often a BMI between 30 and 35 kg/m^2^. Along the same line with previously discussed, influences of the stage of diabetes this could also be of influence here. However, no multicollinearity was present in our statistical multivariable model, i.e. there was no interaction between BMI, diabetes duration and use of DPP4 inhibitors. Taken together, the relation of DPP4 inhibitors and magnesium needs confirmation and further exploration in future studies.

Loop and thiazide diuretics are well known to lower magnesium concentrations. It is therefore quite remarkable that there was no association between the use of diuretics and magnesium in our cohort. This might be explained by the fact that also potassium-sparing diuretics, that lower magnesium excretion, were included in the same group of diuretics. Also the limited number of patients could be involved here.

It should be emphasised that serum magnesium concentrations were used as a surrogate for total magnesium concentration. This has pros and cons. Magnesium is the second most prevalent intracellular cation; this fraction accounts for ~45% of the total body magnesium. Extracellular magnesium accounts for 1% of the total body magnesium content, with the remainder in the skeleton. Approximately 55% of extracellular magnesium is free, 30% is associated with proteins, and primarily albumin and 15% is complexed with anions. Furthermore, it should be emphasised that non-DM related causes of hypomagnesaemia such as decreased intake or malabsorption, use of over the-counter-medication including proton pump inhibitors, increased gastro-intestinal loss e.g. due to diarrhoea or medication cannot be excluded in the present cohort. Furthermore, although most currently used blood glucose lowering medications were analysed in this study, data on sodium-glucose co-transporter-2 (SGLT) inhibitors lack, because at the time of study this class of medication was not yet available for clinical uses in the Netherlands. This may become a concern in future as SGLT2 inhibitors may increase urinary magnesium excretion [24].

Other limitations of the present study should be mentioned as well. Due to the cross-sectional nature of the study, we are unable to prove causality between magnesium concentrations and disease status. This also precludes predictions on the development of the comorbidities. For 45% of potentially eligible participants with T2DM we were unable to match blood samples with clinical data. In addition our cohort is from a single geographic region and consists predominantly (98.8%) of Caucasians, limiting generalisation of our data. On the other hand, strengths of the study include the large cohort size and the extensive clinical and biochemical characterisation of our participants.

Taken together, in this well-defined cohort of 929 outpatients with T2DM treated in primary care, increasing age, diabetes duration, BMI, HbA1c, the use of metformin, sulfonylurea derivatives, DPP4 inhibitors and RAAS blockers were significantly associated with lower serum magnesium concentrations, while LDL cholesterol and creatinine were associated with higher concentrations. Overall, hypomagnesaemia was present in 9.6% of all participants. This percentage is remarkably lower than found in previous studies and may suggest that the prevalence of hypomagnesaemia among persons with T2DM treated in primary care is lower than previously suggested.
